# Quantification of Neighborhood-Level Social Determinants of Health in the Continental United States

**DOI:** 10.1001/jamanetworkopen.2019.19928

**Published:** 2020-01-29

**Authors:** Marynia Kolak, Jay Bhatt, Yoon Hong Park, Norma A. Padrón, Ayrin Molefe

**Affiliations:** 1 Center for Spatial Data Science, Searle Chemistry Laboratory, University of Chicago, Chicago, Illinois; 2 Center for Health Innovation, American Hospital Association, Chicago, Illinois

## Abstract

**Importance:**

An association between social and neighborhood characteristics and health outcomes has been reported but remains poorly understood owing to complex multidimensional factors that vary across geographic space.

**Objectives:**

To quantify social determinants of health (SDOH) as multiple dimensions across the continental United States (the 48 contiguous states and the District of Columbia) at a small-area resolution and to examine the association of SDOH with premature mortality within Chicago, Illinois.

**Design, Setting, and Participants:**

In this cross-sectional study, census tracts from the US Census Bureau from 2014 were used to develop multidimensional SDOH indices and a regional typology of the continental United States at a small-area level (n = 71 901 census tracts with approximately 312 million persons) using dimension reduction and clustering machine learning techniques (unsupervised algorithms used to reduce dimensions of multivariate data). The SDOH indices were used to estimate age-adjusted mortality rates in Chicago (n = 789 census tracts with approximately 7.5 million persons) with a spatial regression for the same period, while controlling for violent crime.

**Main Outcomes and Measures:**

Fifteen variables, measured as a 5-year mean, were selected to characterize SDOH as small-area variations for demographic characteristics of vulnerable groups, economic status, social and neighborhood characteristics, and housing and transportation availability at the census-tract level. This SDOH data matrix was reduced to 4 indices reflecting advantage, isolation, opportunity, and mixed immigrant cohesion and accessibility, which were then clustered into 7 distinct multidimensional neighborhood typologies. The association between SDOH indices and premature mortality (defined as death before age 75 years) in Chicago was measured by years of potential life lost and aggregated to a 5-year mean. Data analyses were conducted between July 1, 2018, and August 30, 2019.

**Results:**

Among the 71 901 census tracts examined across the continental United States, a median (interquartile range) of 27.2% (47.1%) of residents had minority status, 12.1% (7.5%) had disabilities, 22.9% (7.6%) were 18 years and younger, and 13.6% (8.1%) were 65 years and older. Among the 789 census tracts examined in Chicago, a median (interquartile range) of 80.4% (56.3%) of residents had minority status, 10.2% (8.2%) had disabilities, 23.2% (10.9%) were 18 years and younger, and 9.5% (7.1%) were 65 years and older. Four SDOH indices accounted for 71% of the variance across all census tracts in the continental United States in 2014. The SDOH neighborhood typology of extreme poverty, which is of greatest concern to health care practitioners and policy advocates, comprised only 9.6% of all census tracts across the continental United States but characterized small areas of known public health crises. An association was observed between all SDOH indices and age-adjusted premature mortality rates in Chicago (*R*^2^ = 0.63; *P* < .001), even after accounting for violent crime and spatial structures.

**Conclusions and Relevance:**

The modeling of SDOH as multivariate indices rather than as a singular deprivation index may better capture the complexity and spatial heterogeneity underlying SDOH. During a time of increased attention to SDOH, this analysis may provide actionable information for key stakeholders with respect to the focus of interventions.

## Key Points

**Question:**

How do social determinants of health vary across multiple dimensions and geographic space?

**Findings:**

In this cross-sectional study of 71 901 census tracts with approximately 312 million persons across the continental United States, multivariate social determinants of health measures were reduced to 4 indices reflecting advantage, isolation, opportunity, and mixed immigrant cohesion and accessibility and were clustered into 7 neighborhood typologies that included an extreme poverty group. Social determinants of health indices were associated with premature mortality rates in Chicago, Illinois.

**Meaning:**

The use of multidimensional geospatial approaches to quantify social determinants of health rather than the use of a singular deprivation index may better capture the complexity and spatial heterogeneity underlying these determinants.

## Teaser

This cross-sectional study uses data from the US Census Bureau and a multidimensional geospatial approach to quantify social determinants of health across the continental United States and to examine the association of social determinants of health with premature mortality in Chicago, Illinois.

## Introduction

The consequences of social determinants of health (SDOH) increasingly dominate public health discussions in the United States, as population health outcomes have not kept pace with those of other developed nations despite higher per-person spending for medical services.^1,2,3^ An increased understanding of SDOH could be used to better connect patients with relevant social services in clinical contexts and could target vulnerable populations with health-improving social policies and programs while also addressing affordability.^4,5^ Health policy frameworks that directly address the underlying social and behavioral determinants of health are now encouraged to promote improvements in population health outcomes and cost savings.^4^ However, more finely quantifying these determinants at scale remains a challenge.

Social determinants of health are defined by the World Health Organization as the conditions in which people are born, grow, live, work, and age.^6^ Despite this complex and nuanced description of SDOH, the phenomena are often represented solely by socioeconomic indicators, such as income and education. Social determinants of health indicators, such as income, are associated with greater life expectancy in the United States; however, these associations are complex and may change based on underlying area characteristics and health behaviors.^7^ While these variables are associated with health outcomes, they are also likely to be associated with each other, resulting in issues of multicollinearity and presenting challenges for meaningful interpretation.^8^

Index-based approaches have also emerged to serve as proxies to assess the consequences of SDOH and have incorporated various methods. The area deprivation index (ADI) by Singh et al,^9,10,11^ which was extended by Kind et al,^12^ focused on socioeconomic disadvantage and the differing dimensions of poverty. The North Carolina Institute of Public Health and the Carolinas HealthCare System developed a conceptual model–driven SDOH index that includes additional dimensions of health-related social needs, such as food accessibility; however, this index has not yet been validated against actualized health outcomes.^8^ Other conceptual frameworks incorporate a wide range of indicators with a broad range of applications for data collection, clinical use, and research. Proxies used to assess SDOH remain varied and unstandardized and may obfuscate the underlying factors involved in differential health outcomes. These approaches may also overlook the geographic heterogeneity of the US population or the ways in which uniquely characterized neighborhoods, such as retirement communities or diverse urban immigrant enclaves, complicate a unidimensional application of SDOH.

We addressed these challenges by implementing a cross-sectional multivariate analysis of SDOH components using a principal component and regionalization cluster analyses. In a cross-sectional view, SDOH may have more explanatory power for health outcomes than do the differences associated with changes in medical care or technology.^8^ We hypothesized that actual SDOH outcomes may vary from existing conceptual models, although they should remain associated with health outcomes in meaningful ways. In the first phase, we developed a multidimensional SDOH data matrix for the continental United States (the 48 contiguous states and the District of Columbia) at the census-tract level for 2014. Census tracts are small-area geographic regions defined by the US Census Bureau for analyzing populations that approximate neighborhoods containing between 2500 and 8000 persons. We then developed a multidimensional index based on the results of a principal component analysis and imported the findings into spatially sensitive clusters or typologies of SDOH across the continental United States. In the second phase, we associated the SDOH multidimensional index with age-adjusted premature mortality within Chicago, Illinois, a large metropolitan city with recognized challenges in health outcomes and social and economic disparities.

## Methods

### Population and Spatial Scale

In this cross-sectional multivariate analysis, the first phase of the study included all populated census tracts of the continental United States (n = 71 901), with a total observed population of approximately 312 million persons based on census estimates. To create the index, we used only the continental states to preserve neighboring relationships across tracts regardless of state boundary. In the second phase, we validated the index using a subset of Chicago with 789 census tracts that included approximately 7.5 million persons. We chose the census tract as the spatial scale of our analysis to estimate small-area variations at the neighborhood level. A total of 6908 of 71 901 census tracts (9.6%) were in the extreme poverty group (Table 1). A waiver of informed consent was granted by the institutional review board of the University of Chicago because this study used nonidentifiable public data sets that did not constitute human subjects research. Data analyses were conducted between July 1, 2018, and August 30, 2019 (eMethods in the Supplement). We used the Strengthening the Reporting of Observational Studies in Epidemiology (STROBE) checklist for cross-sectional analysis to guide the reporting of results.

**Table 1.** Summary Statistics of Social Determinants of Health Typologies for United States and Chicago Tracts^a^

Variable	Median (IQR) [CV]													
	Rural Affordable		Vibrant Urban Core		Suburban Affordable		Extreme Poverty		Multilingual Working		Suburban Affluent		Sparse Areas	
	US	Chicago	US	Chicago	US	Chicago	US	Chicago	US	Chicago	US	Chicago	US	Chicago
Census tracts, No.	19 512	28	4619	230	14 017	33	6908	249	6352	141	17 811	102	2682	8
Population, thousands	76 282	106.4	77 530	841.9	73 473	140.2	22 662	787.4	31 304	594.4	19 602	403.7	11 131	32.1
Ethnic/racial minority, %	15.3 (27.2) [1.0]	97.0 (19.5) [0.2]	47.4 (44.6) [0.5]	40.5 (34.0) [0.5]	31.4 (36.8 ) [0.7]	68.0 (47.0) [0.5]	78.9 (43.6) [0.4]	98.0 (2.0) [0.1]	86.7 (20.1) [0.2]	89.0 (18.0) [0.1]	16.7 (21.9) [0.8]	33.0 (24.5) [0.5]	12.9 (19.8) [1.0]	28.5 (72.2) [0.8]
Aged ≥65 y, %	16.9 (5.7) [0.3]	19.9 (5.3) [0.2]	10.2 (7.9) [0.6]	8.7 (7.9) [0.7]	9.7 (4.9) [0.4]	8.9 (3.7) [0.3]	10.3 (5.8) [0.4]	10.6 (7.2) [0.5]	8.6 (5.3) [0.5]	7.5 (3.6) [0.4]	15.7 (6.2) [0.3]	12.4 (6.4) [0.4]	32.9 (15.5) [0.4]	32.2 (12.4) [0.3]
Aged ≤18 y, %	22.0 (4.9) [0.2]	20.6 (6.7) [0.3]	14.7 (11.8) [0.5]	13.7 (9.5) [0.5]	27.2 (5.3) [0.2]	26.7 (5.1) [0.1]	27.0 (8.2) [0.3]	27.8 (8.7) [0.2]	28.3 (8.4) [0.2]	28.4 (7.0) [0.2]	20.7 (5) [0.2]	21.0 (4.8) [0.2]	12.3 (6.9) [0.5]	14.1 (5.3) [0.4]
Disability, %	16.6 (5.1) [0.2]	16.0 (4.2) [0.2]	9.1 (6.2) [0.5]	7.0 (6.0) [0.6]	9.0 (4.4) [0.3]	8.0 (2.0) [0.2]	17.2 (7.0) [0.3]	15.0 (5) [0.3]	10.1 (5.0) [0.4]	8.0 (3.0) [0.3]	9.8 (4.1) [0.3]	9.0 (4.0) [0.4]	20.5 (8.9) [0.4]	22.5 (8.5) [0.3]
No high school diploma, %	14.5 (9.5) [0.4]	11.5 (12.2) [0.5]	10.9 (14.2) [0.8]	7.0 (12.0) [0.9]	7.8 (8.1) [0.7]	16.0 (16.0) [0.6]	21.9 (12.2) [0.4]	21.0 (12.0) [0.4]	35.0 (17.8) [0.3]	37.0 (17.0) [0.3]	5.8 (5.3) [0.7]	9.0 (9.0) [0.6]	10.4 (10.8) [0.8]	14.5 (7.8) [0.6]
Limited English proficiency, %	0.6 (2.1) [1.6]	0 (3.0) [1.6]	4.5 (10.1) [1.1]	2.5 (8.0) [1.2]	1.6 (3.6) [1.1]	8.0 (12.0) [0.8]	1.2 (4.7) [1.4]	0 (1.0) [2.1]	19.7 (12.0) [0.4]	25.0 (8.0) [0.3]	2.1 (2.4) [1.3]	5.0 (8.0) [0.9]	0.8 (2.7) [2.0]	15.0 (11.0) [1.1]
Single parent, %	8.5 (5.3) [0.4]	9.7 (4.2) [0.4]	5.7 (8.5) [0.9]	4.0 (6.0) [0.9]	10.0 (6.9) [0.5]	11.2 (6.9) [0.4]	19.8 (9.4) [0.4]	22.1 (11.4) [0.4]	14.2 (9.2) [0.5]	14.1 (8.7) [0.4]	5.3 (4.2) [0.6]	5.2 (4.5) [0.6]	3.4 (4.1) [0.8]	2.3 (3.1) [1.7]
Living in poverty, %	16.1 (10.4) [0.5]	17.5 (6.4) [0.3]	18.4 (18.0) [0.7]	16.4 (13.1) [0.5]	9.1 (9.7) [0.7]	13.2 (10.1) [0.6]	37.0 (15.9) [0.3]	38.5 (17.7) [0.3]	25.7 (16.6) [0.4]	24.7 (11.8) [0.4]	6.5 (6.3) [0.8]	8.1 (8.8) [0.6]	12.2 (12.1) [0.8]	12.8 (16.2) [0.7]
Per capita income, $	22 382 (6273) [0.2]	21 773 (4874) [0.2]	28 959 (2433) [0.7]	41 546 (32 931) [0.5]	27 955 (11 403) [0.3]	23 302 (9323) [0.3]	14 545 (5512) [0.3]	14 210 (6398) [0.3]	15 648 (7082) [0.3]	15 108 (6075) [0.3]	36 383 (15 323) [0.4]	32 961 (17 268) [0.4]	28 383 (15 291) [0.5]	27 310 (2819) [0.6]
Unemployed, %	9.5 (5.7) [0.4]	18.0 (7.5) [0.3]	8.5 (6.4) [0.5]	7.0 (7.0) [0.6]	7.2 (5.1) [0.5]	11.0 (5.0) [0.3]	19.0 (10) [0.4]	26.0 (12.0) [0.3]	11.6 (6.7) [0.4]	12.0 (5.0) [0.3]	6.1 (3.9) [0.5]	8.0 (4.8) [0.4]	9.5 (7.3) [0.7]	10.5 (6.8) [0.6]
Uninsured, %	14.4 (8.2) [0.4]	15.2 (5.2) [0.3]	12.5 (11.2) [0.6]	12.2 (12.6) [0.6]	10.9 (9.3) [0.6]	13.7 (13.3) [0.5]	18.1 (9.5) [0.4]	19.7 (7.9) [0.3]	29.6 (12.1) [0.3]	29.9 (7.6) [0.2]	7.1 (6.3) [0.6]	11.2 (11.0) [0.6]	11.8 (10.4) [0.6]	7.0 (8.4) [0.8]
Renter, %	25.0 (8.9) [0.5]	26.0 (10.5) [0.5]	73.0 (25.0) [0.2]	63.0 (15.0) [0.2]	25.0 (25.0) [0.7]	19.0 (19.0) [0.6]	60.0 (24.0) [0.3]	67.0 (24.0) [0.3]	51.0 (32.0) [0.4]	57.0 (19.0) [0.3]	23.0 (24) [0.7]	35.0 (25.0) [0.5]	22.0 (23.0) [0.8]	48.5 (34.5) [0.5]
Rent burden, %	12.0 (8.9) [0.5]	13.5 (11.8) [0.6]	26.8 (27.9) [0.8]	26.0 (25.5) [0.7]	9.2 (10.7) [0.8]	11.0 (12.0) [0.7]	18.6 (18.1) [0.7]	23.0 (25.0) [0.8]	13.0 (16.1) [0.9]	20.0 (17.0) [0.6]	15.6 (13.1) [0.6]	18.5 (12.5) [0.5]	16.6 (14.1) [0.6]	31.5 (25.0) [0.6]
Crowded housing, %	1.6 (2.5) [1.0]	1.0 (3.0) [1.2]	3.4 (6.4) [1.1]	1.0 (3.0) [1.3]	1.8 (3.1) [1.1]	3.0 (4.0) [0.9]	3.0 (4.4) [1.0]	4.0 (4.0) [0.8]	13.1 (10.8) [0.6]	10.0 (9.0) [0.6]	0.7 (1.9) [1.4]	1.0 (2.0) [1.4]	0.6 (1.9) [1.5]	0.5 (2.0) [1.4]
No vehicle, %	5.5 (5.7) [0.7]	15.2 (8.6) [0.4]	27.8 (35.0) [0.7]	30.6 (19.3) [0.4]	2.6 (3.8) [1.0]	7.1 (6.8) [0.6]	21.7 (17.6) [0.6]	36.8 (17.7) [0.3]	9.3 (11.0) [0.9]	17.6 (9.3) [0.4]	3.8 (5.1) [0.9]	10.4 (6.2) [0.5]	6.4 (9.8) [1.2]	39.0 (24.0) [0.5]
Advantage score^b^	0.5 (1.6) [3.4]	−0.8 (0.9) [−0.9]	−0.8 (3.1) [−2.5]	0.3 (3.3) [−48.9]	0.9 (2.1) [2.3]	−0.7 (2.8) [−3.2]	−3.1 (2.3) [−0.5]	−4.4 (2.5) [−0.4]	−4.0 (2.8) [−0.5]	−4.4 (2.3) [−0.4]	2.1 (1.4) [0.6]	0.9 (2.2) [1.7]	1.7 (2.3) [1.3]	0.9 (2.7) [306.6]
Mobility score^c^	−0.7 (1.4) [−0.9]	−1.4 (1.2) [−0.8]	−0.2 (1.4) [−3.6]	0.2 (1.3) [−19.0]	1.1 (1.0) [0.6]	1.2 (0.5) [0.4]	−1.2 (1.6) [−0.9]	−1.6 (1.3) [−0.7]	1.6 (1.4) [0.6]	1.6 (1.1) [0.5]	0.4 (1.0) [1.9]	0.7 (0.8) [1.0]	−2.5 (1.6) [−0.6]	−3.1 (1.6) [−0.4]
Opportunity score^d^	−0.7 (0.7) [−0.8]	0 (0.8) [−13.1]	2.6 (1.5) [0.4]	2.8 (1.3 ) [0.3]	−0.6 (0.7) [−0.9]	−0.5 (0.6) [−1.0]	−0.4 (1.3) [−2.7]	0.1 (1.2) [4.6]	0.1 (1.4) [5.2]	0.7 (1.0) [1.0]	0.4 (0.9) [1.3]	1.0 (0.9) [0.7]	0.6 (1.5) [1.8]	1.9 (1.4) [0.4]
MICA score^e^	−0.4 (0.7) [1.4]	0 (0.7) [6.7]	0.70 (1.3) [−1.4]	1.00 (1.1) [−1.0]	0.5 (0.7) [−1.0]	0 (0.9) [−2.7]	1.3 (1.2) [−0.6]	2.2 (1.6) [−0.5]	−1.5 (1.4) [0.6]	−1.2 (1.0) [0.6]	0.1 (0.7) [−9.2]	0.2 (1.1) [−5.0]	−1.6 (1.0) [0.6]	−0.9 (0.8) [0.6]

Abbreviations: CV, coefficient of variation; IQR, interquartile range; MICA, mixed immigrant cohesion and accessibility index.

^a^ Summary statistics are for each variable within each typology for US and Chicago tracts. Summaries for each typology can be considered with respect to the median estimates shown in Table 2.

^b^ First primary component; Advantage indicates socioeconomic advantage index.

^c^ Second primary component; Mobility indicates limited mobility index.

^d^ Third primary component; Opportunity indicates urban core opportunity index.

^e^ Fourth primary component.

### Data Definitions

#### Social Determinants of Health

**Figure 1.**
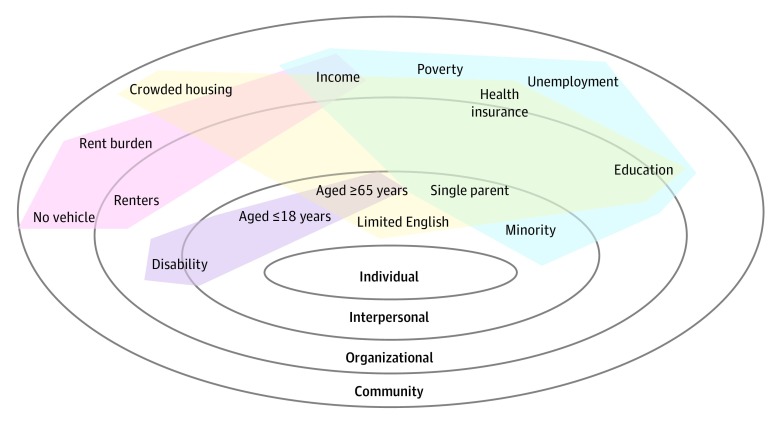
Socioecological Model of Health and Social Determinants of Health Indices Social determinants of health indicators are mapped onto different spheres of the socioecological model of health adapted from Bronfenbrenner.^18^ Principal components include indicators for each index. Blue shading indicates socioeconomic advantage index, purple shading indicates limited mobility index, red shading indicates urban core opportunity index, and yellow shading indicates mixed immigrant cohesion and accessibility index.

Existing SDOH conceptual frameworks acknowledge the underlying indicators that are likely important factors in differential health outcomes. We reviewed multiple conceptual SDOH frameworks to identify indicators that could be measured in an empirical model at scale, including the Danaher, World Health Organization, and California Department of Public Health frameworks and the American Heart Association and American College of Cardiology SDOH guidelines and measures that are common in healthy places models.^13,14,15,16,17^ Fifteen variables were chosen to capture small-area variations in economic status, social and neighborhood characteristics, housing and transportation availability, and demographic characteristics of vulnerable groups. We stratified these indicators across 3 broad topics that are common to multiple SDOH frameworks, representing social, economic, and physical environments. To facilitate more meaningful interpretation between these concepts, we also mapped each indicator selected onto the socioecological model of health,^18^ which was adapted to include individual, interpersonal, organizational, and community spheres (Figure 1).

Demographic features of socioeconomically vulnerable groups include areas with high proportions of older adults (aged ≥65 years), persons with minority status, and persons with disabilities, which are all features associated with differentiating health outcomes and behaviors.^18,19,20^ Additional social disadvantages, such as limited English language proficiency^21,22^ and single-parent households,^23,24^ are also associated with health outcomes. Vulnerable age groups, single-parent households, persons with limited English proficiency, and persons with minority status were mapped to the interpersonal sphere, which reflected localized social networks, family, and cultures. Disability status by neighborhood proportion was mapped across interpersonal and organizational spheres, reflecting complex interactions within social networks and organizations that are factors in social inclusion.^25^

We chose standard economic indicators with known associations with health outcomes, including poverty,^15,16,26^ income,^7,27^ unemployment,^28,29^ education,^4,27,30,31,32^ and health insurance status.^33,34^ Educational level and health insurance status are included as service environments in some SDOH conceptual frameworks.^15^ Educational level and health insurance status thus crossed both organizational and community spheres in our socioecological model adaptation. We included poverty, income, and unemployment status in the community sphere, which reflected structural societal properties.

The physical grouping included housing and transportation measures. Studies have also documented associations between housing status and health risks^35,36,37,38^ as well as the consequences of vehicle access on health behaviors.^39^ To serve as a proxy for housing characteristics, we included the proportion of renters, rent burden (ie, more than 30% of income used for rent payments), and crowded housing conditions (ie, occupied housing units consisting of more people than rooms). Crowded housing and rent burden were included in the community sphere, which reflected wider patterns of housing availability and cost, while the proportion of renters was identified across organizational and community spheres. To serve as a proxy for vehicle access, we included households with no vehicle in the community sphere as a structural component that may reflect wider geographic patterns.

We limited the analysis to data available as continuous variables across continental tracts in the United States for the period of interest. All variables were derived from the 2014 American Community Survey 5-year mean. The associated proxies and the data sources are presented in Table 2.

**Table 2.** Social Determinants of Health Data^a^

Variable	Median (IQR) [CV]	
	US (n = 71901)	Chicago (n = 789)
Ethnic/racial minority^b^	27.(247.1) [0.8]	80.4 (56.3) [0.4]
Aged ≥65 y	13.6 (8.1) [0.5]	9.5 (7.1) [0.6]
Aged ≤18 y	22.9 (7.6) [0.3]	23.2 (10.9) [0.4]
Disability^c^	12.1 (7.5) [0.5]	10.2 (8.2) [0.5]
No high school diploma^d^	11.4 (13.5) [0.8]	17.4 (18.7) [0.7]
Limited English proficiency^e^	1.3 (4.6) [1.7]	3.3 (13.5) [1.2]
Single parent^f^	8.4 (7.9) [0.7]	11.1 (14.1) [0.8]
Living in poverty	13.3 (15.7) [0.8]	21.9 (21.8) [0.6]
Per capita income, $	25 174 (14 511) [0.5]	21 232 (19 127) [0.7]
Unemployed^g^	8.5 (6.7) [0.6]	12.9 (12.8) [0.7]
Uninsured^h^	12.6 (11.5) [0.6]	17.9 (12.8) [0.5]
Renter	31 (32) [0.6]	58 (27) [0.4]
Rent burden^i^	13.4 (13.3) [0.8]	21.7 (21.0) [0.7]
Crowded housing^j^	1.8 (3.7) [1.5]	3.3 5.9) [1.00]
No vehicle	5.5 (8.8) [1.3]	24.1 (22.9) [0.6]

Abbreviations: CV, coefficient of variation; IQR, interquartile range.

^a^ Data are estimates from the 2014 American Community Survey 5-year mean.

^b^ Defined as persons of all racial/ethnic ancestries with the exception of white, non-Hispanic ancestry.

^c^ Persons in the civilian noninstitutionalized population.

^d^ Persons aged 25 years or older.

^e^ Persons aged 5 years or older.

^f^ Households with children aged less than 18 years.

^g^ Civilians aged 16 years and older.

^h^ Persons in the total civilian noninstitutionalized population.

^i^ Renters paying more than 30% of their household income for rent.

^j^ Defined as occupied housing units consisting of more people than rooms.

#### Health Outcomes and Violent Crime

To estimate associations between social determinants of health and health outcomes for a subset of data, the mortality rate at the census-tract level was used for the Chicago, for which we had sufficiently high-quality direct measurements of premature mortality. These data were obtained from the Chicago Department of Public Health. Premature mortality (ie, death before age 75 years) was measured by years of potential life lost, aggregated to a 5-year mean (2009-2013) and calculated as an age-adjusted rate at the census-tract level.

We accessed crime data from the Chicago data portal and obtained 2014 data from a subset of violent crime categories, including battery, assault, robbery, and homicide. We then used the available geocodes to translate crimes as spatial point data, and we spatially joined intersecting census tracts and aggregated data by tract using the spdep package (version 1.1-3) in the R software environment (R Foundation for Statistical Computing). To obtain a violent crime rate, we calculated the total number of violent crimes per population of associated census tract.

### Statistical Analysis

All statistical tests were 2-sided with a significance level of *P* = .05. All statistical analyses were performed using the R software environment (version 3.6.1), GeoDa (version 1.14.0), and GeoDaSpace (version 1.1) statistical software (Center for Spatial Data Science).

#### Exploratory Factor Analysis

We implemented a principal component analysis of the SDOH matrix. In a principal component analysis, multiple variables are reduced to core components, with each component orthogonal (ie, not correlated) by construction to preserve the most information given by their variances. This approach is similar to the original construction of the Singh ADI, which used principal component analysis for index development.^9^ We extended the Singh methodology with a more complex conceptual model of SDOH that incorporated additional variables reflecting multiple dimensions of health, and we further minimized variables that were likely to be associated with each other (eg, families classified as living in poverty and households with poverty levels of more than 150%). Because the first index approximated socioeconomic disadvantage, we examined its association with the Singh ADI index using a Spearman correlation coefficient. We also developed a composite SDOH index by summing principal component scores.

We implemented the principal component analysis using the singular-value decomposition method and initial variable standardization, and we used the Kaiser criterion (ie, components with eigenvalues of less than 1.0 were excluded) to determine the final number of components to retain. In accordance with literature standards, we used 0.30 variable loading for the component as the cutoff for the for determining the dominant variables within each principal component. Final components, which together accounted for most of the variance in all 15 SDOH variables, were visualized as SD maps.

#### Regionalization Analysis

We conducted a dimension-reducing clustering analysis to decompose tracts into typologies that had similar SDOH characteristics. Similar to principal component analyses, this clustering analysis is a machine learning technique that uses unsupervised algorithms to reduce dimensions of multivariate data. To examine regional typologies, we collapsed the 4 dimensions associated with SDOH into a single dimension to give insight into the spatial heterogeneity of outcomes and examine the reasons that different results occurred in different places. The most important aspects of the construction were that each typology had similar attributes within its own grouping and that each typology was distinct. The attributes used for the analysis were distinct principal components that reflected unique phenomena (orthogonal by construction), which provided more meaningful results than would loading the algorithm with dozens of highly correlated variables. Region types are useful for a variety of methodological needs, such as the need to use 1 dimension of data to compare and contrast neighborhoods across the continental United States or to identify similar areas for matching analyses in quasi-experimental studies.

Consistent with work that has used algorithms to define areas with both geographic and socioeconomic components,^40,41,42^ we implemented multivariate regionalization techniques that joined areas based on attribute similarity and a minimal degree of spatial connectivity. We inputted dominant principal components into a k-means clustering analysis to identify k groups of tracts with similar SDOH characteristics. Because census tracts are nonphysical boundaries, in which residents are able to easily cross barriers and affect nearby tracts, it was important to account for spatial proximity between tracts. Geometric centroids of census tracts were thus given a 0.10 weighting within the k-means analysis to enforce a minimal but explicit spatial sensitivity. The k-means analysis was performed for 2 through 15 clusters for 71 901 tracts using the Arthur and Vassilvitskii^43^ k-means^++^ procedure. The number of clusters that retained the lowest change in the proportion of between to total within-cluster sum of squares was chosen as an optimum. The typologies of the final clusters were summarized with descriptive statistics and visualized as an interactive map.

#### Regression Analysis

We estimated associations between premature mortality rates in Chicago using the 4 indices derived from the dominant principal components while controlling for the violent crime rate. The indices were used as input to retain the greatest information rather than as k-means clusters because transforming the continuous principal components to discrete clusters invariably results in the loss of information. First, we estimated a linear regression model using ordinary least squares (OLS) estimation. A spatial weight matrix was constructed for the census tract data set using second-order queen contiguity, assigning bordering neighbors (up to 2 census tracts away) for each tract. We implemented a spatial autoregressive (SAR) model, incorporating spatially lagged mortality and 2-stage least squares estimation.^44^

## Results

Among the 71 901 census tracts (n = 312 million persons) examined across the continental United States, a median (interquartile range [IQR]) of 27.2% (47.1%) of residents had minority status, 12.1% (7.5%) had disabilities, 22.9% (7.6%) were 18 years and younger, and 13.6% (8.1%) were 65 years and older. Among the 789 census tracts (n = 7.5 million persons) examined in Chicago, a median (IQR) of 80.4% (56.3%) of residents had minority status, 10.2% (8.2%) had disabilities, 23.2% (10.9%) were 18 years and younger, and 9.5% (7.1%) were 65 years and older. Additional baseline and result summary statistics are shown in Table 1 and Table 2.

### Principal Component Analysis

Four principal components—the socioeconomic advantage index, the limited mobility index, the urban core opportunity index, and the mixed immigrant cohesion and accessibility index—met the Kaiser criterion for inclusion. Together, they accounted for 71% of the variance in the 15 SDOH variables across all census tracts in the continental United States. Principal component analysis scores had a mean of 0 by construction and were not further standardized to retain their original distributions (box and whisker plots of each component available in eFigure 1 in the Supplement).

The first principal component, the socioeconomic advantage index, accounted for 40.0% of the total variance and was dominated by socioeconomic status factors, including poverty, low high school graduation rates, minority status, proportion of uninsured persons, and number of single-parent households. We adjusted for cardinality so that areas with low proportions of socioeconomic disadvantage were positive and labeled this component the socioeconomic advantage index. This index could thus serve as a proxy for multidimensional poverty or socioeconomic disadvantage.

While other principal components reflected additional dimensions of socioeconomic disadvantage, the first index was principally characterized by the classic measures of socioeconomic factors, such as poverty, minority status, and educational level. The variables of minority status (−0.32), no high school diploma (−0.34), living in poverty (−0.32), and uninsured (−0.31) were associated with each other, with higher absolute variable loadings in the same direction (eTable 1 and eTable 2 in the Supplement), thus reflecting a unique phenomenon. This index’s association between minority status and poverty may reflect the role that racial segregation has played in perpetuating environments that are associated with health disparities.^45,46^ Comparison with the ADI index at the tract level indicated a Spearman rank correlation of 0.59 between aggregated (as means) ADI national ranks and aggregated (as means) socioeconomic advantage index ranks. When identifying indicators that were important factors of this component in our adapted socioecological model, we found that this phenomenon was present across all spheres of health (interpersonal, organizational, and community), as mapped in Figure 1.

The second principal component, the limited mobility index, accounted for 13.4% of the variance and was dominated by areas with high proportions of older adults and persons with disabilities; these areas were negatively associated with the presence of children. Dominant principal component variable loadings for older adults (−0.41) and persons with disabilities (−0.58) were in opposite directions to children (0.34), as shown in eTable 1 in the Supplement. Such neighborhoods may reflect uniquely vulnerable, isolated populations with limited mobility; thus, we labeled this component the limited mobility index. This index was focused within the inner scales of our adapted socioecological model of health, which was primarily associated with interpersonal and organizational dimensions.

We used the terms mobility and isolation to systematically reflect the complex interactions between aging, disability, and transportation. Older adults are more likely to remain in their homes, make fewer trips when they do leave home, and travel shorter distances, reflecting age-specific mobility issues even after controlling for confounding factors.^47^ These reduced mobility issues are factors in both resource accessibility and social isolation needs. Older adults are also at greater risk of losing critical components of their social ties as they age, making access to social capital of great importance for their health—and in the United States, both social capital and community support of older adults have decreased over time.^48^ The utility of this geographic approach is illustrated by ongoing research to identify areas with a high concentration of older populations to improve transportation policy, social service facilities, and planning.^49,50^

Neighborhoods with a greater number of older adults and persons with disabilities may be at greater risk of mobility issues and social isolation, which poses unique challenges when considering SDOH and health outcomes. For example, accessibility to health care and food resources in areas disproportionately populated by persons with less mobility owing to age and disabilities would require different assumptions about minimum distance traveled and transportation modality. These areas may represent neighborhoods with more senior housing or retirement and aging communities in disadvantaged areas that are experiencing economic decline. The limited mobility index is thus most useful when considered alongside the other indices. For example, low-income, socially isolated older adults are especially sensitive to environmental events, such as heat emergencies.

The third principal component, the urban core opportunity index, which accounted for 9.6% of the variance, included highly urbanized populations with high opportunity and corresponding high costs. High-scoring areas of this index, as defined by dominant variable loadings of the principal component analysis, were associated with high per capita income (0.36), high proportion of renters (0.38), high rent burden (0.38), and households without a vehicle (0.43; eTable 1 and eTable 2 in the Supplement). These areas also had fewer children. This index was focused on the outer spheres of our adapted socioecological model of health, which were associated with organizational and community dimensions. These areas were characterized by their compact geographies, dense urban centers, and strong economies, which were likely factors in the high local incomes.

The walkability and diversity of the areas further reflected characteristics of new urbanism and postmodern cities, which have been discussed in urban analysis literature.^51,52^ Although these areas may attract residents who seek housing that is close to their jobs, the high rent burden may be a disadvantage of that location choice. These areas thus have high job opportunities and well-established transportation infrastructures, but they also have a high cost of living that may have negative consequences for vulnerable individuals residing within them.

The fourth principal component, the mixed immigrant cohesion and accessibility index, which accounted for 8.1% of the variance, was characterized by mostly immigrant or multilingual groups with traditional family structures and multiple accessibility stressors. Areas with low scores on the mixed immigrant cohesion and accessibility index, as defined by dominant variable loadings of the principal component analysis, had high proportions of families with limited English proficiency (−0.41), older adults (−0.42), and crowded housing (−0.31) as well as a lack of health insurance (−0.30), lower high school graduation rates (−0.31), and fewer single parent households (0.32; eTable 1 and eTable 2 in the Supplement). Like the socioeconomic advantage index, this principal component was mapped across all spheres of health (interpersonal, organizational, and community), as shown in Figure 1.

This index also reflected dimensions of disadvantage, but it did so in a slightly different way than the socioeconomic advantage index. Vulnerable immigrant communities were represented, but the communities had unique patterns regarding accessibility and social cohesion. We viewed access as a complex topic with multiple dimensions.^53,54^ Because it was not clear why these residents lacked these specific resources, we assumed it was related to a structural failure in accessibility, such as affordability, adequacy of services, or awareness of services. At the same time, the higher rates of older adults and children and the smaller number of single-family households suggested more cohesive family units, stronger interpersonal ties, and social connections that may have served as protective factors. Ongoing research seeks to better understand the role of language and social connection development, which are factors associated with immigrant health.^55^

The immigrant experience of acculturative stress and the decrease in family cohesion over time pose further challenges to achieving better health outcomes.^56^ Scores on the mixed immigrant cohesion and accessibility index were negative and low in multilingual typologies (−1.50 in the United States and −1.20 in Chicago) that were also likely to have negative and low scores on the socioeconomic advantage index (−4.0 in the United States and −4.4 in Chicago), reflecting socioeconomic stressors (Table 1) (eTable 2 in the Supplement).

**Figure 2.**
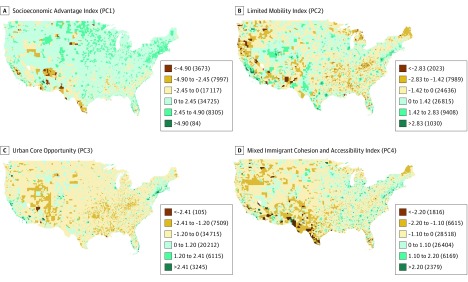
Social Determinants of Health Indices for Continental United States A, Socioeconomic advantage index (PC1). B, Limited mobility index (PC2). C, Urban core opportunity index (PC3). D, Mixed immigrant cohesion and accessibility index (PC4). Index scores are provided for each interval, with total number of corresponding census tracts indicated in parentheses. Data classification corresponds to SDs, with brown shades indicating deviations below the mean and blue shades indicating deviations above the mean. PC1 indicates first primary component; PC2, second primary component; PC3, third primary component; and PC4, fourth primary component.

Mapped indices, using SD data classification, indicate spatially heterogeneous patterns across the continental United States (Figure 2) and Chicago (eFigure 2 in the Supplement). Areas of greatest socioeconomic disadvantage were found in small and dense tracts of cities throughout the country from Los Angeles, California, to Baltimore, Maryland, in addition to larger, more sparsely populated tracts across multiple Native American reservations across South Dakota, Wyoming, and Arizona. The southern border of Texas and a wide area through the southern United States were also likely to have more socioeconomic disadvantage, especially in comparison with the northern United States. A more heterogeneous pattern was found in the mobility-related isolation index; some areas with high proportions of older adults and persons with disabilities dominated the southern United States, Appalachia, southern and western sections of Chicago, and areas in parts of Florida and Phoenix, Arizona. The areas with the highest urban opportunity scores were limited to tightly bounded tracts in areas such as the centers of New York City and San Francisco, California, and the northern Chicago bordering the lake. Low outliers of the fourth principal component index reflected multilingual communities from southern Texas to the western and northwestern sides of Chicago, all with more traditional family structures and less advantageous accessibility characteristics.

### Regionalization Analysis

A cumulative SDOH index was calculated by adding all 4 component index scores, weighting each by their proportional variance from the principal component analysis. A cumulative SDOH index mapped across the continental United States (eFigure 3 in the Supplement) indicates a pattern similar to that of the socioeconomic advantage index, reflecting the dominant weighting of this index. In eFigure 4 in the Supplement, a scatter plot of the cumulative index and premature mortality rates in Chicago indicates that census tracts with higher mortality rates had lower index scores, reflecting higher vulnerability. To keep the maximum possible data, it is preferable to retain each index separately, although this singular index can be useful for exploratory analysis and comparison purposes.

The k-means analysis identified 7 optimal clusters, each corresponding to unique neighborhood typologies (eFigure 5 in the Supplement). We used both the summary statistics themes and the geographic patterns of the typologies to inform the names of the regional categories. The 7 optimal clusters of SDOH in the study area were categorized as (1) rural affordable, characterized by higher proportions of older adults and persons with disabilities, moderate income levels, and few persons living in crowded housing or without vehicles; (2) suburban affordable, characterized by high vehicle ownership, few renters, and a high proportion of children; (3) suburban affluent, characterized by high income, a high proportion of children, few persons without vehicles, and low poverty; (4) vibrant urban core, characterized by high income, a high proportion of renters, and few children; (5) extreme poverty, characterized by a high proportion of residents with minority status, low income, high poverty, and high unemployment; (6) multilingual working, characterized by a high proportion of residents with minority status, low English proficiency, low income, and low unemployment; and (7) sparse areas, characterized by a high proportion of older adults and generally located within national or state forests, parks, or other natural areas.

The regional typologies were complex. To impose as minimal a construct as possible, we used the minimum number of qualifiers to describe each category. We had not anticipated that these typologies would serve as proxies for different rural, suburban, and urban types, but we found this characteristic helpful for interpretation. However, we caution that these category names are approximations and generalizations. Social determinants of health clusters or typologies are visualized across the United States in eFigure 4 in the Supplement, with summary statistics shown in Table 1. Results from an analysis of variance highlighted the significance of the cluster attribute value compared with all other tracts. As shown in eTable 3 in the Supplement, each indicator mean for each typology census tract was significantly different from all other census tract means. Index and clustering results can be explored in the form of a table or an interactive map at the SDOH atlas website,^57^ with the magnification tool enabled for meaningful exploration. To further facilitate interpretation of these results, we have provided eTable 2 in the Supplement, which includes data regarding the direction and magnitude of indices across each regional typology for the continental United States and Chicago. For example, the socioeconomic advantage index was an order of magnitude lower in the extreme poverty and multilingual working group typologies compared with all other census tracts.

At a macro level, rural affordable areas were present across the country. Suburban affluent areas were clustered in the northeastern area of the United States and scattered across wider regions across the northern United States. Sparse areas with more natural, preserved environments were found in Maine and the southwestern, northwestern, and northern sections of Michigan and Wisconsin. Vibrant urban core areas were not visible at a macro level because they were generally small, densely populated census tracts contained within enclaves of large urban environments, such as New York City, Los Angeles, and San Francisco (SDOH typologies within Chicago are shown in eFigure 5 in the Supplement). Although clusters of extreme poverty were visible in the southern United States from Mississippi to Alabama, most of these census tracts were likewise smaller and visible when magnified to neighborhood levels.

When considering how typologies vary across space together, the continental United States can be viewed as a complex patchwork of nuanced SDOH characteristics. Some areas were characterized by their tremendous spatial heterogeneity across SDOH neighborhood typologies, such as Florida, which was not obviously dominated by any specific group. Other areas were characterized by their relative spatial homogeneity, such as Oklahoma, which mainly included rural and suburban affordable areas. Notably, all 7 geographic typologies were present in Chicago, which is a spatially heterogenous city known for its neighborhoods and socioeconomic disparities (Table 1).

### Regression Analysis

We found that in Chicago, more than 60% of the variation in premature mortality at the neighborhood level was associated with SDOH dimensions alone, even after accounting for violent crime and underlying spatial structures. An association was observed between all SDOH indices and age-adjusted premature mortality in Chicago (OLS adjusted *R*^2^ = 0.61 and SAR adjusted *R*^2^ = 0.63; *P* < .001). Increased rates of premature mortality were associated with the principal components of less advantage (OLS estimate, −0.18; *P* < .001 and SAR estimate, −0.12; *P* < .001), less mobility (OLS estimate, −0.49; *P* < .001 and SAR estimate, −0.37; *P* < .001), less opportunity (OLS estimate, −0.29; *P* < .001 and SAR estimate, −0.17; *P* < .001), lower scores on the mixed immigrant cohesion and accessibility index (OLS estimate, −0.09; *P* = .003 and SAR estimate, −0.07; *P* = .01), and higher crime (OLS estimate, 0.50; *P* = .005 and SAR estimate, 0.34; *P* < .001). Results are shown in Table 3, with software output included in eResults in the Supplement. Although census tracts with high immigrant or multilingual populations were associated with better health outcomes in Chicago despite the city’s more disadvantaged socioeconomic characteristics, this association was inverted from positive to negative when the limited mobility index was added to the regression analysis. The sensitivity of the mixed immigrant cohesion and accessibility index may have thus reflected a nuanced and complicated underlying pattern. Low accessibility measures may have outweighed the protective, socially cohesive factors characteristic of communities of recent immigrants that mitigated health outcomes (Table 3).

**Table 3.** Results of Regression Analysis^a^

Measure	OLS Estimate (SE)	*P* Value	SAR Estimate (SE)	*P* Value
Advantage^b^	−0.179 (0.033)	<.001	−0.118 (0.031)	<.001
Mobility^c^	−0.490 (0.030)	<.001	−0.370 (0. 031)	<.001
Opportunity^d^	−0.289 (0.026)	<.001	−0.168 (0.028)	<.001
MICA^e^	−0.085 (0.030)	.003	−0.070 (0.027)	.01
Violent crime	0.496 (0.108)	.005	0.341 (0.088)	<.001
Spatially lagged YPLL	NA	NA	0.396 (0.150)	<.001
*R^2^*	0.614	NA	0.643	NA
Adjusted *R^2^*	0.612	NA	0.627	NA
Mean squared error	0.036	NA	0.032	NA

Abbreviations: Advantage, socioeconomic advantage index; MICA, mixed immigrant cohesion and accessibility index; Mobility, limited mobility index; NA, not applicable; OLS, ordinary least square; Opportunity, urban core opportunity index; SAR, spatial autoregressive model; YPLL, years of potential life lost.

^a^ The regression analysis included 789 observations. The SAR model results are shown with maximum likelihood estimation. The generalized method of moments estimation results are available in eResults in the Supplement.

^b^ First primary component.

^c^ Second primary component.

^d^ Third primary component.

^e^ Fourth primary component.

## Discussion

The design, implementation, and evaluation of effective policies at the local, state, and federal levels to improve health outcomes can be improved through a deeper understanding of the complexities of social and economic disparities. In this study, we aimed to decompose and disentangle these complexities using publicly available data, and we found that most variation in data across all census tracts in the continental United States can be quantified by 4 core components that reflect socioeconomic advantage, limited mobility, urban core opportunity, and mixed immigrant cohesion and accessibility. Notably, while socioeconomic disadvantage was the dominant factor in this variation, social and neighborhood environment characteristics together accounted for almost the same amount of variation, underscoring the complexity and nuance of place-based SDOH indicator differences. These multidimensional indices can be further summarized as 7 SDOH clusters or typologies that serve as proxies for urban, suburban, and rural neighborhood groups as well as areas of both extreme wealth and poverty. When considering multiple dimensions of health, the compounding of socioeconomic disadvantage, vulnerable population distribution, low opportunity, and low accessibility may increase disparities.

Areas of extreme poverty not only reflect disadvantage but may also signal wider patterns of community deinvestment and infrastructure breakdown that together suggest consequences for population-level health outcomes. The extreme poverty SDOH neighborhood typology of greatest concern to health care practitioners and policy advocates comprised only 9.6% of all census tracts across the continental United States but characterized small areas of known public health crises, including lead contamination of drinking water in Flint, Michigan,^58,59^ lead exposure among children in some parts of Chicago,^60^ and parasitic worm outbreaks associated with a lack of sanitation in rural regions of Alabama.^61^ These public health crises have had disproportionate consequences in disadvantaged communities and have often resulted from breakdowns in the basic infrastructures necessary for health. Our SDOH neighborhood typology identified and differentiated these areas in a nationwide, standardized manner and thus may be useful for further research and investigation and may serve as an approach to prioritize resources, interventions, and future investments.

Our findings confirm and extend the literature associating SDOH indicators with health outcomes, including policy reviews based on both clinical research and population-based studies, which have hypothesized that 60% of early deaths in the United States are associated with nonclinical factors.^2,30^ Our approach allowed us to present a framework for understanding the intrinsically complex associations between education, poverty, and health outcomes, and it is well-suited for policy design, as it makes explicit factors of neighborhood environment characteristics that may be important factors in all policies designed to address SDOH. Our Chicago case study findings are consistent with those of Singh et al,^9,10,11^ which indicated that socioeconomic indicators could account for almost half of the total variance in health outcomes.^7,8,9^ Educational level, another important factor in our first component index, has likewise been associated with almost half of the deaths in working-age populations in the United States.^31^ Our analysis confirms that the use of intercorrelated indicators of SDOH likely introduces issues of endogeneity in analysis (as hypothesized by Fuchs^8^) and may obfuscate the underlying phenomenon of socioeconomic advantage and overlook the further complexity of neighborhood patterns. The ADI and similar approaches served as proxies for the first principal component (the socioeconomic advantage index) in our analysis but may have missed the additional social and neighborhood environment characteristics that together may be equally important. By using indicators of disadvantage alone, researchers may further underestimate the consequences of additional social and built environment characteristics.

Empirical assessment of the SDOH conceptual model is difficult because the complex causal pathways remain unknown, and challenges exist in identifying which factors are associated with worse health outcomes and in understanding the causes of the causes.^62^ Our findings suggest that existing models, which focus on traditional measures of socioeconomic disadvantage (poverty, educational level, and minority status) that are generally associated with each other, may not account for additional population-level patterns that can interact with each other or serve as their own unique indicators. More specifically, we hope that by quantifying the distinct dimensions of SDOH, researchers can better understand why SDOH interacts with health outcomes differently in different places. The interactions between socioeconomic disadvantage and limited mobility areas provide an example, as areas with higher rates of poverty and larger numbers of older adults and persons with minority status are likely more vulnerable than areas with either dimension on its own.

### Limitations

Our study had several limitations. Notably, our analysis was an in-depth empirical exploration of SDOH indicators, their interactions, and their associations with premature mortality within a limited period. Because of the potentially unmeasured factors and unknown associations reported in SDOH literature, the results should be interpreted broadly and with caution. Multiple additional factors could and should be introduced in future work to determine the sensitivity of results, including an exploration of outcomes across different races and ethnicities to better approximate distinct associations. The data we used only allowed for cross-sectional analyses, which may have introduced the risk of missing changes (or the lack thereof) in socioeconomic patterns and associated health outcomes over time. Our analysis was performed at an aggregate level and reflected population means rather than individual phenomena. The documented problems of ecological fallacy^63^ and modified areal units^64^ suggest that caution should be used when interpreting or extrapolating our findings on an individual level.

In addition, because we wanted to retain patterns in both attribute and geographic data dimensions, more precise measurement of uncertainty across all variables in all census tracts was not straightforward. Regionalization has been used as a tool to reduce uncertainty in census data, as aggregating tracts to regions with similar characteristics may increase sampling sizes and reduce margins of error.^65^ We attempted to minimize uncertainty by using tract-level estimates and clustering techniques. However, more measurement tool innovation is needed to account for uncertainty across the methods implemented in this study. Finally, owing to data availability, we limited our analysis to premature mortality as a health outcome. Social determinants of health indicators have different associations with differing health outcomes and should be further investigated at small-area and individual-level resolutions.

## Conclusions

We believe a multidimensional instrumentation for quantifying SDOH provides greater understanding of the ways in which underlying small-area structures are associated with health outcomes. Although socioeconomic advantage may be an important factor in the overall direction of health outcomes, those outcomes remain slightly varied across different populations in different places.^4^ A place-based approach is focused on distilling factors associated with later-stage disease outcomes, which are far more costly, substantial, and unique. By focusing on these outcomes and working backward, factors can be identified that may lead to an understanding of why these diseases progress and what we can do to prevent them, a process we call the place-based framework.

Our empirical, exploratory approach sought to better understand and connect concepts across SDOH conceptual frameworks and the socioecological view of health, extending existing research and providing careful attention to the association between variables, geographic data science methods that are sensitive to spatial patterns, and the development of multidimensional indices that are useful for planning and practice. Further research is needed to estimate the consequences of the different dimensions of SDOH on health outcomes across multiple geographic places. Stakeholders can use these results, in conjunction with their own data, to assist in identifying areas of targeted intervention to improve health outcomes. Marginalized populations may experience place-based inequities that are associated with the occurrence of poor health. Given their high consequences on health care spending, these inequities may also be key to implementing substantial improvements in health care quality and value. Interventions targeting high-risk subpopulations may deliver direct benefits and produce large improvements at the local, regional, and national levels.

## Notes

Supplement. **eMethods.** Spatial Scale, Data and Software, YPLL Data Definition, and Regional Typologies **eResults.** OLS and SAR Estimations **eFigure 1.** Raw Distributions of Principal Component Scores **eFigure 2.** SDOH Indices for Chicago **eFigure 3.** Cumulative SDOH Index Across Continental United States **eFigure 4.** Association Between Cumulative SDOH Factors and Years of Potential Life Lost at Tract Level for Chicago **eFigure 5.** SDOH Neighborhood Typology **eTable 1.** Principal Component Analysis Overview **eTable 2.** SDOH Indices, US Typology, and Chicago Typology **eTable 3.** Average and Standard Deviation for Each Variable of Interest Across All US Tracts **eReferences.**
