# Supplementary material for: Quantification of Neighborhood-Level Social Determinants of Health in the Continental United States
Source: JAMA Netw Open. 2020 Jan 29;3(1):e1919928. doi: 10.1001/jamanetworkopen.2019.19928 (PMC6991288; doi:10.1001/jamanetworkopen.2019.19928)
Supplement: Supplement. — eMethods. Spatial Scale, Data and Software, YPLL Data Definition, and Regional Typologies eResults. OLS and SAR Estimations eFigure 1. Raw Distributions of Principal Component Scores eFigure 2. SDOH Indices for Chicago eFigure 3. Cumulative SDOH Index Across Continental United States eFigure 4. Association Between Cumulative SDOH Factors and Years of Potential Life Lost at Tract Level for Chicago eFigure 5. SDOH Neighborhood Typology eTable 1. Principal Component Analysis Overview eTable 2. SDOH Indices, US Typology, and Chicago Typology eTable 3. Average and Standard Deviation for Each Variable of Interest Across All US Tracts eReferences. [file jamanetwopen-3-e1919928-s001.pdf]

Supplementary Online Content

Kolak M, Bhatt J, Park YH, Padrón NA, Molefe A. Quantification of neighborhood-level social determinants of health in the continental United States. *JAMA Netw Open*. 2020;3(1):e1919928. doi:10.1001/jamanetworkopen.2019.19928

eMethods. Spatial Scale, Data and Software, YPLL Data Definition, and Regional Typologies

eResults. OLS and SAR Estimations

eFigure 1. Raw Distributions of Principal Component Scores

eFigure 2. SDOH Indices for Chicago

eFigure 3. Cumulative SDOH Index Across Continental United States

eFigure 4. Association Between Cumulative SDOH Factors and Years of Potential Life Lost at Tract Level for Chicago

eFigure 5. SDOH Neighborhood Typology

eTable 1. Principal Component Analysis Overview

eTable 2. SDOH Indices, US Typology, and Chicago Typology

eTable 3. Average and Standard Deviation for Each Variable of Interest Across All US Tracts

eReferences.

This supplementary material has been provided by the authors to give readers additional information about their work.

© 2020 Kolak M et al. *JAMA Network Open.*

eMethods. Spatial Scale, Data and Software, YPLL Data Definition, and Regional Typologies

Spatial Scale. Our use of the tract is in contrast to the Kind et al[1] extension of the Areal Deprivation Index, which was computed at the block group level. Recent geodemographic research indicates that census data quality varies substantially across spatial scale, and retains a high margin of error below tract-level.[2,3] As such, the tract is the finest resolution available that facilitates reliable analysis and linkage to non-decadal census socioeconomic variables.

Data and Software. Most census variables were available in cleaned format as part of the Social Vulnerability Index (SVI) database.[4] Two variables, proportion of renters and rent burden, were not available in cleaned format, and extracted from the United States Census Bureau data portal site, American FactFinder. The final data was cleaned, merged, and combined with spatial data formats using R and GeoDa.[5] Data cleaning was done in R. The principal component analysis, static mapping, and final spatial data file preparation was performed in GeoDa.[36] The interactive map was developed with Carto and made available with all data results at https://SDOHAtlas.github.io. The k-means cluster was recorded for each census tract in GeoDa, joined to the master census tract file, and made interactive through Carto web-based software. Both regressions were conducted using GeoDa and GeoDaSpace statistical software.

YPLL Data Definition. Years Potential Life Lost (YPLL): Summary measure of years lost due to premature death per 100,000 population below age 75. In contrast to mortality measures, YPLL emphasizes the effect of premature mortality on a population. YPLL is the sum of the differences between a predetermined end point (e.g., age 75 or average life expectancy) and the ages of death for those who died before that end point, divided by the total population at or below that end point, and multiplied by 100,000. For example, people who die before age 75 are defined as having lost some potential years of life. Because of the way in which YPLL is calculated, this measure gives more weight to a death the earlier it occurs. For instance, although most deaths occur at advanced ages, more years of potential life are lost for deaths among younger age groups, especially for certain causes, such as HIV/AIDS, accidents, homicide and suicide.

© 2020 Kolak M et al. *JAMA Network Open.*

Regional Typologies. The “rural” tracts are generally in geographic areas generally regarded as rural due to low population density and correspond to high percentage of car ownership associated with rurality. Because poverty, rent burden, crowded housing are all low, we consider these areas generally affordable with respect to other areas. “Sparse areas” were an interesting tract subgroup that occurred within or near rural areas, often next to wide expanses of forested parks or natural spaces. “Suburban” tracts generally occurred between “rural” and “urban” tracts geographically, also had high car ownership, and more children overall. We distinguish these otherwise similar “suburban” categories by differences in their economic characteristics; “affluent” areas had higher income rates, lower unemployment, and lower poverty. While this typology is useful for comparisons and exploring unique geographical characteristics of the data, region typologies are not as useful for regression analyses. Because region types are categorical and retain less information than the components used to generate them, we use the raw indices to retain maximum information.

© 2020 Kolak M et al. *JAMA Network Open*.

eResults. OLS and SAR Estimations

OLS Estimation

```
SUMMARY OF OUTPUT: ORDINARY LEAST SQUARES
-----------------------------------------
Data set            :ChicagoTracts_nonulls.dbf
Weights matrix      :File: ChicagoTracts_nonulls-q2.gal
Dependent Variable  :       YPLLS                Number of Observations:         791
Mean dependent var  :      0.0000                Number of Variables   :           6
S.D. dependent var  :      1.0000                Degrees of Freedom    :         785
R-squared           :      0.6141
Adjusted R-squared  :      0.6116
Sum squared residual:     304.883                F-statistic           :    249.8119
Sigma-square        :       0.388                Prob(F-statistic)     :  1.433e-159
S.E. of regression  :       0.623                Log likelihood        :    -745.322
Sigma-square ML     :       0.385                Akaike info criterion :    1502.645
S.E of regression ML:      0.6208                Schwarz criterion     :    1530.685

------------------------------------------------------------------------------------
            Variable     Coefficient       Std.Error     t-Statistic     Probability
------------------------------------------------------------------------------------
            CONSTANT       0.8370367       0.1509168       5.5463463       0.0000000
                PC1L      -0.1785854       0.0313683      -5.6931771       0.0000000
                PC2L      -0.4900934       0.0283431     -17.2914277       0.0000000
                PC3L      -0.2886548       0.0258513     -11.1659613       0.0000000
                PC4L      -0.0844521       0.0280446      -3.0113500       0.0026843
           VCRIMRT14       0.4957240       0.0884098       5.6071152       0.0000000
------------------------------------------------------------------------------------

-----------------------------------------
Data set            :ChicagoTracts_nonulls.dbf
Dependent Variable  :       YPLLS                Number of Observations:         791
Mean dependent var  :      0.0000                Number of Variables   :           6
S.D. dependent var  :      1.0000                Degrees of Freedom    :         785
R-squared           :      0.6141
Adjusted R-squared  :      0.6116
Sum squared residual:     304.883                F-statistic           :    249.8119
Sigma-square        :       0.388                Prob(F-statistic)     :  1.433e-159
S.E. of regression  :       0.623                Log likelihood        :    -745.322
Sigma-square ML     :       0.385                Akaike info criterion :    1502.645
S.E of regression ML:      0.6208                Schwarz criterion     :    1530.685

White Standard Errors
------------------------------------------------------------------------------------
            Variable     Coefficient       Std.Error     t-Statistic     Probability
```

© 2020 Kolak M et al. *JAMA Network Open.*

CONSTANT	0.8370367	0.1834241	4.5633949	0.0000058
PC1L	-0.1785854	0.0325934	-5.4791956	0.0000001
PC2L	-0.4900934	0.0298433	-16.4222189	0.0000000
PC3L	-0.2886548	0.0256680	-11.2456913	0.0000000
PC4L	-0.0844521	0.0298547	-2.8287694	0.0047919
VCRIMRT14	0.4957240	0.1084886	4.5693674	0.0000057

© 2020 Kolak M et al. *JAMA Network Open.*

SAR Estimation

```
SUMMARY OF OUTPUT: MAXIMUM LIKELIHOOD SPATIAL LAG (METHOD = FULL)
-----------------------------------------------------------------
Data set            :ChicagoTracts_nonulls.dbf
Weights matrix      :File: ChicagoTracts_nonulls-q2.gal
Dependent Variable  :       YPLLS                Number of Observations:         791
Mean dependent var  :      0.0000                Number of Variables   :           7
S.D. dependent var  :      1.0000                Degrees of Freedom    :         784
Pseudo R-squared    :      0.6420
Spatial Pseudo R-squared:  0.6286
Sigma-square ML     :       0.358                Log likelihood        :    -719.297
S.E of regression   :       0.598                Akaike info criterion :    1452.593
                                                 Schwarz criterion     :    1485.306

------------------------------------------------------------------------------------
            Variable     Coefficient       Std.Error     z-Statistic     Probability
------------------------------------------------------------------------------------
            CONSTANT       0.5725160       0.1495877       3.8272934       0.0001296
                PC1L      -0.1180986       0.0312163      -3.7832332       0.0001548
                PC2L      -0.3696625       0.0310094     -11.9209818       0.0000000
                PC3L      -0.1679154       0.0281293      -5.9694175       0.0000000
                PC4L      -0.0710792       0.0269111      -2.6412609       0.0082598
           VCRIMRT14       0.3405106       0.0876771       3.8836896       0.0001029
             W_YPLLS       0.3957278       0.0514206       7.6959067       0.0000000

----------
SUMMARY OF OUTPUT: SPATIAL TWO STAGE LEAST SQUARES
--------------------------------------------------
Data set            :ChicagoTracts_nonulls.dbf
Weights matrix      :File: ChicagoTracts_nonulls-q2.gal
Dependent Variable  :       YPLLS                Number of Observations:         791
Mean dependent var  :      0.0000                Number of Variables   :           7
S.D. dependent var  :      1.0000                Degrees of Freedom    :         784
Pseudo R-squared    :      0.6425
Spatial Pseudo R-squared:  0.6278

------------------------------------------------------------------------------------
            Variable     Coefficient       Std.Error     z-Statistic     Probability
------------------------------------------------------------------------------------
            CONSTANT       0.5466742       0.1516461       3.6049343       0.0003122
                PC1L      -0.1121894       0.0318154      -3.5262645       0.0004215
                PC2L      -0.3578972       0.0341379     -10.4838812       0.0000000
                PC3L      -0.1561200       0.0323024      -4.8330809       0.0000013
                PC4L      -0.0697728       0.0269863      -2.5854919       0.0097240
           VCRIMRT14       0.3253473       0.0888500       3.6617571       0.0002505
```

© 2020 Kolak M et al. *JAMA Network Open.*

```
            W_YPLLS       0.4343876       0.0678943       6.3980018       0.0000000
------------------------------------------------------------------------------------
Instrumented: W_YPLLS
Instruments: W_PC1L, W_PC2L, W_PC3L, W_PC4L, W_VCRIMRT14

DIAGNOSTICS FOR SPATIAL DEPENDENCE
TEST                           MI/DF       VALUE           PROB
Anselin-Kelejian Test             1           0.238          0.6255
================================ END OF REPORT =====================================
```

```
REGRESSION
----------
SUMMARY OF OUTPUT: SPATIAL TWO STAGE LEAST SQUARES
--------------------------------------------------
Data set            :ChicagoTracts_nonulls.dbf
Weights matrix      :File: ChicagoTracts_nonulls-q2.gal
Dependent Variable  :       YPLLS                Number of Observations:         791
Mean dependent var  :      0.0000                Number of Variables   :           7
S.D. dependent var  :      1.0000                Degrees of Freedom    :         784
Pseudo R-squared    :      0.6425
Spatial Pseudo R-squared:  0.6278

White Standard Errors
------------------------------------------------------------------------------------
            Variable     Coefficient       Std.Error     z-Statistic     Probability
------------------------------------------------------------------------------------
            CONSTANT       0.5466742       0.1773093       3.0831675       0.0020481
                PC1L      -0.1121894       0.0333441      -3.3645917       0.0007666
                PC2L      -0.3578972       0.0368508      -9.7120535       0.0000000
                PC3L      -0.1561200       0.0337074      -4.6316288       0.0000036
                PC4L      -0.0697728       0.0282811      -2.4671180       0.0136205
           VCRIMRT14       0.3253473       0.1038843       3.1318225       0.0017372
             W_YPLLS       0.4343876       0.0801084       5.4224990       0.0000001
------------------------------------------------------------------------------------
Instrumented: W_YPLLS
Instruments: W_PC1L, W_PC2L, W_PC3L, W_PC4L, W_VCRIMRT14

DIAGNOSTICS FOR SPATIAL DEPENDENCE
TEST                           MI/DF       VALUE           PROB
Anselin-Kelejian Test             1           0.238          0.6255
================================ END OF REPORT =====================================
```

© 2020 Kolak M et al. *JAMA Network Open.*

eFigure 1. Raw Distributions of Principal Component Scores

PC1: Socioeconomic Advantage, PC2: Isolation-Related Mobility,
PC3: Urban Core Opportunity, and PC4: Mixed Immigrant Cohesion and Accessibility

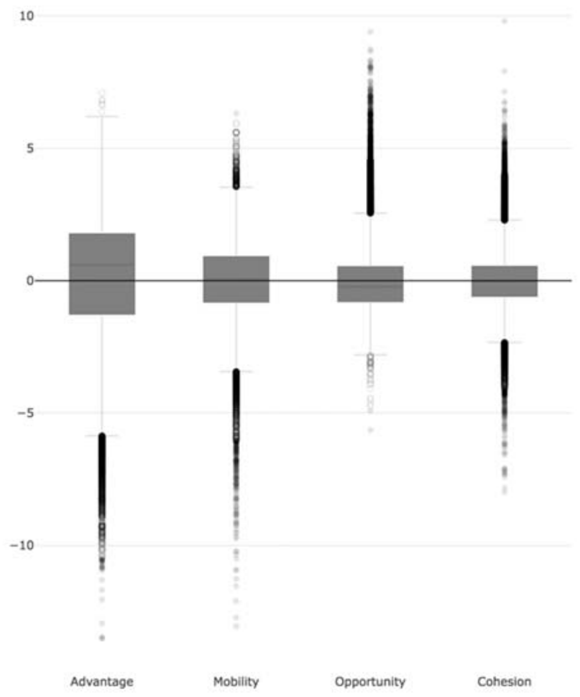

© 2020 Kolak M et al. *JAMA Network Open*.

eFigure 2. SDOH Indices for Chicago

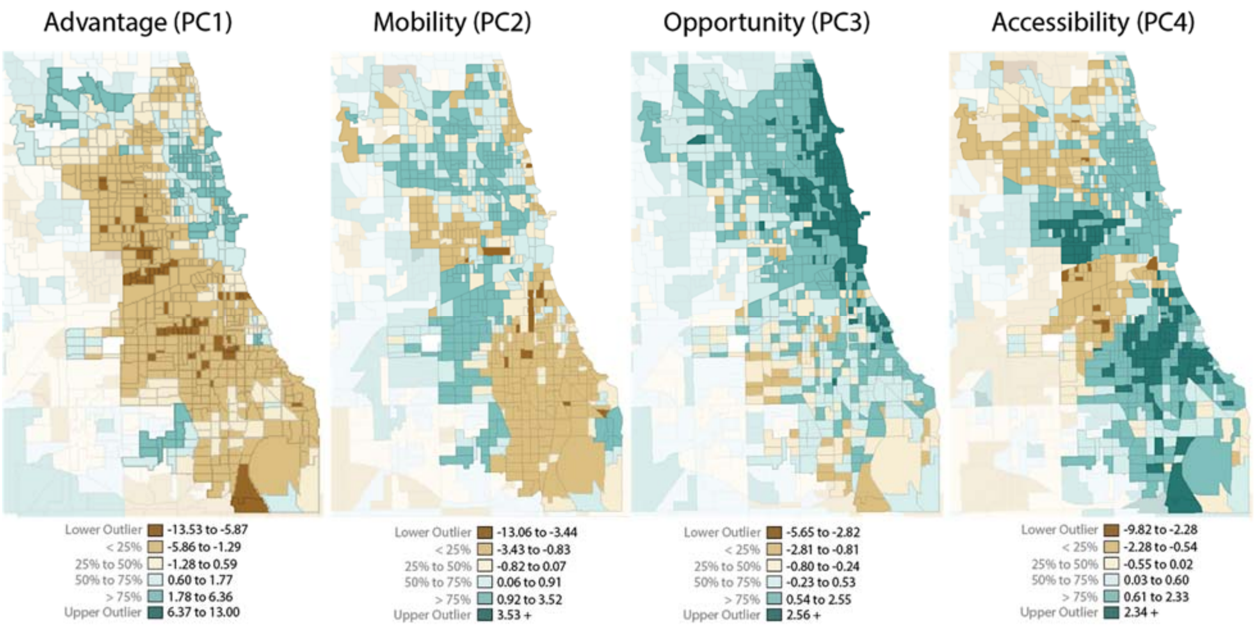

Hinge = 1.5

© 2020 Kolak M et al. *JAMA Network Open*.

eFigure 3. Cumulative SDOH Index Across Continental United States

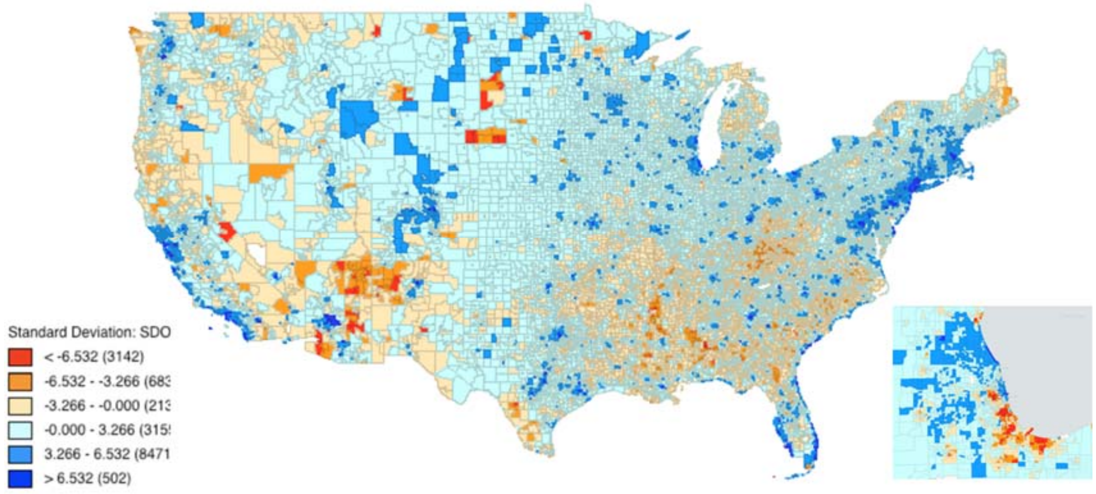

Inset: Chicago Area.

© 2020 Kolak M et al. *JAMA Network Open*.

eFigure 4. Association Between Cumulative SDOH Factors and Years of Potential Life Lost at Tract Level for Chicago

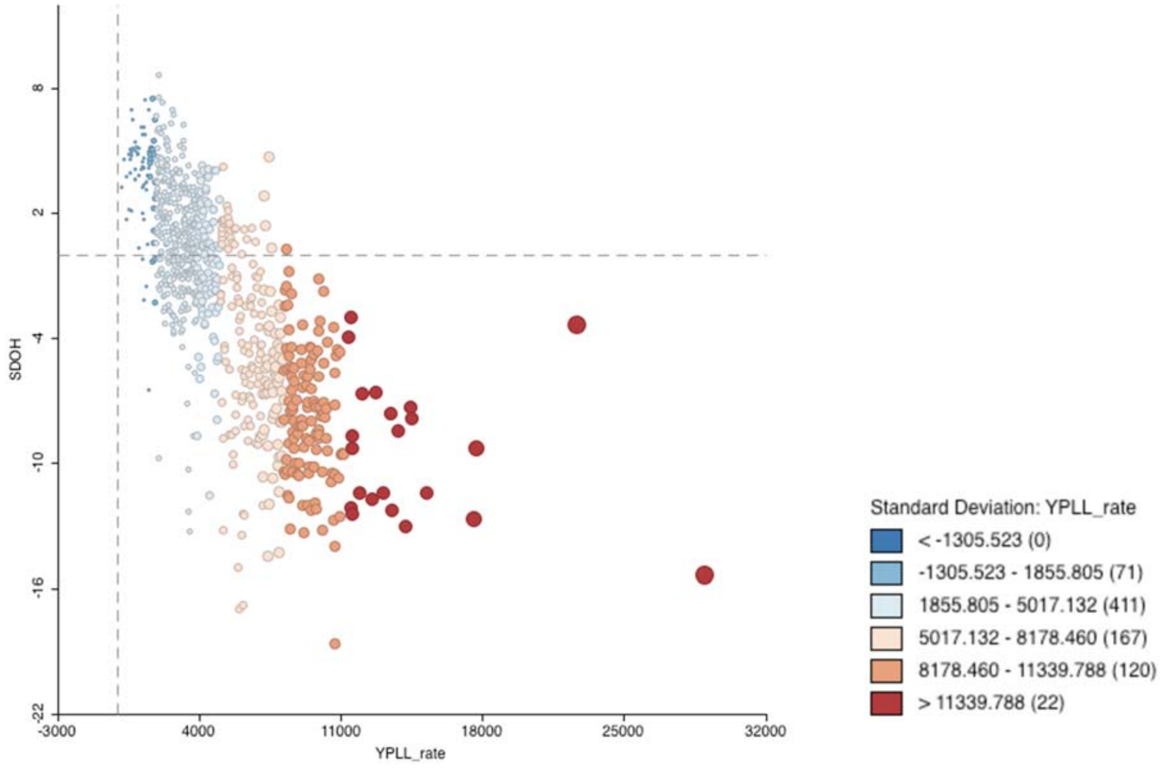

© 2020 Kolak M et al. *JAMA Network Open*.

eFigure 5. SDOH Neighborhood Typology

Seven SDOH neighborhood typologies were optimized across the continental United States, as presented in this screenshot from the SDOH web application generated to visualize results. Chicago detail shown in inset map.

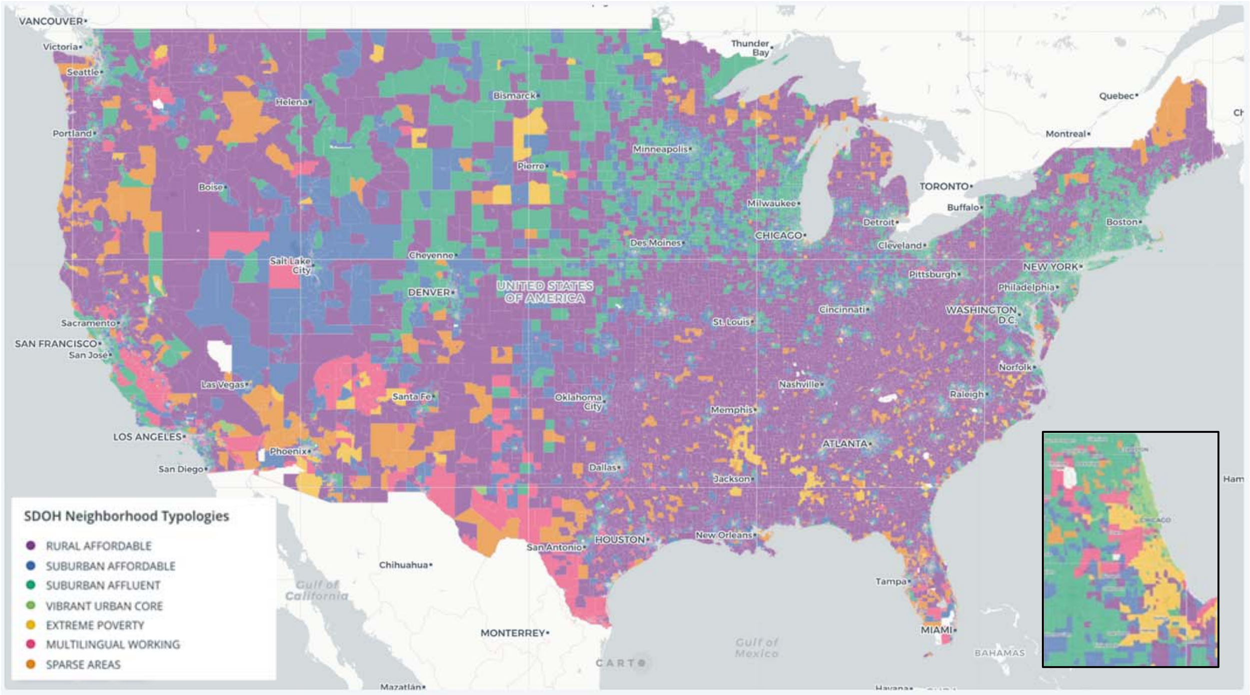

eTable 1. Principal Component Analysis Overview

Variable	PC1	PC2	PC3	PC4
Minority Status	-0.3232	0.1244	0.1293	0.0392
Over Age 65	0.1704	-0.4077	0.0655	-0.4186
Under Age 17	-0.1579	0.3453	-0.4287	0.2178
Disabled	-0.0862	-0.5773	-0.2383	-0.1669
No Highschool	-0.3442	-0.0188	-0.0913	-0.3119
Limited English	-0.2739	0.2591	0.2327	-0.4129
Single Parent	-0.2993	0.0258	-0.2197	0.3190
Poverty	-0.3242	-0.2431	-0.0347	0.1463
Per Capita Income	0.2708	0.1643	0.3578	0.0635
Unemployment	-0.2543	-0.2812	-0.1270	0.1969
Uninsured	-0.3082	0.0359	-0.0751	-0.3017
Renter	-0.2740	-0.0712	0.3727	0.2279
Rent Burden	-0.0622	-0.1199	0.3788	0.1268
Crowded Housing	-0.2878	0.2542	0.1135	-0.3142
No Vehicle	-0.2262	-0.2170	0.4258	0.2375

© 2020 Kolak M et al. *JAMA Network Open.*

eTable 2. SDOH Indices, US Typology, and Chicago Typology

The following tables summarize variables dominant in each principal component, as well as direction and magnitude of the indices across region typologies for both US and Chicago. They may facilitate interpretation of findings.

(a) SDOH Indices

PC1	PC2	PC3	PC4
Minority Status (-)	Over Age 65 (-)	Under Age 17 (-)	Over Age 65 (-)
No Highschool (-)	Under Age 17 (+)	Per Capita Income (+)	No Highschool (-)
Single Parent (-)	Disabled (-)	Renter (+)	Single Parent (+)
Poverty (-)		Rent Burden (+)	Limited English (-)
Uninsured (-)		No Vehicle (+)	Uninsured (-)
			Crowded Housing (-)

(b) US Typology (↑, ↓: magnitude < 1, ⇑, ⇓: magnitude > 1)

	Rural Affordable	Vibrant Urban Core	Suburban Affordable	Extreme Poverty	Multilingual Working	Suburban Affluent	Sparse Area
PC1 **Socioeconomic Advantage**	↑	↓	↑	⇓	⇓	⇑	⇑
PC2 **Mobility-Related Isolation**	↓	↓	⇑	⇓	⇑	↑	⇓
PC3 **Urban Opportunity**	↓	⇑	↓	↓	↑	↑	↑
PC4 **Mixed Cohesion & Accessibility**	↓	↑	↑	⇑	⇓	↑	⇓

© 2020 Kolak M et al. *JAMA Network Open.*

(c) Chicago Typology (↑, ↓: magnitude < 1, ⇑, ⇓: magnitude > 1, .: no difference)

	Rural Affordable	Vibrant Urban Core	Suburban Affordable	Extreme Poverty	Multilingual Working	Suburban Affluent	Sparse Area
PC1 **Socioeconomic Advantage**	.	↓	↓	⇓	⇓	↑	↑
PC2 **Mobility-Related Isolation**	⇓	.	⇑	⇓	⇑	↑	⇓
PC3 **Urban Opportunity**	↓	⇑	↓	↑	↑	.	⇑
PC4 **Mixed Cohesion & Accessibility**	↓	.	↑	⇑	⇓	↑	⇓

© 2020 Kolak M et al. *JAMA Network Open.*

eTable 3. Average and Standard Deviation for Each Variable of Interest Across All US Tracts

The following table reports the average and standard deviation for each variable of interest across all US tracts. An ANOVA test of means, comparing each variable within the typology tract against all other tracts, was conducted as well, with p-value reported for each variable. Results should be interpreted with caution because of the Multiple Comparisons problem.

Variable (Unit)	**Rural Affordable**	**Vibrant Urban Core**	**Suburban Affordable**	**Extreme Poverty**	**Multilingual Working**	**Suburban Affluent**	**Sparse Areas**
	Mean (SD)	*Mean (SD)*	*Mean (SD)*	*Mean (SD)*	*Mean (SD)*	*Mean (SD)*	*Mean (SD)*
Census Tracts (n)	19,512	17,811	14,017	6,908	6,352	4,619	2,682
Population (in thousands)	76,282	77,530	73,473	22,662	31,304	19,602	11,131
Minority (%)	22.51	51.60	35.62	70.78	82.42	22.10	20.01
	(21.40)	(26.82)	(24.23)	(26.97)	15.63	(18.63)	(20.39)
	p=0.000	*p=0.000*	*p=0.000*	*p=0.000*	*p=0.000*	*p=0.000*	*p=0.000*
Over Age 65 (%)	17.20	11.00	9.64	10.68	9.24	15.93	36.45
	(4.38)	(6.36)	(3.48)	(4.46)	(4.23)	(5.16)	(15.41)
	p=0.000	*p=0.000*	*p=0.000*	*p=0.000*	*p=0.000*	*p=0.000*	*p=0.000*
Under Age 18 (%)	21.90	14.89	27.73	26.87	28.09	20.24	11.36
	(3.85)	(7.99)	(4.43)	(6.97)	(6.46)	(4.47)	(5.25)
	p=0.000	*p=0.000*	*p=0.000*	*p=0.000*	*p=0.000*	*p=0.000*	*p=0.000*
Disability (%)	17.17	9.87	9.03	17.73	10.64	9.80	22.04
	(4.05)	(5.02)	(3.10)	(5.75)	(3.99)	(3.05)	(9.24)
	p=0.000	*p=0.000*	*p=0.000*	*p=0.000*	*p=0.000*	*p=0.000*	*p=0.000*
No High School (%)	15.68	13.32	9.13	22.76	36.25	6.61	12.73
	(7.03)	(10.75)	(6.26)	(9.25)	(12.58)	(4.63)	(9.61)
	p=0.000	*p=0.000*	*p=0.000*	*p=0.000*	*p=0.000*	*p=0.000*	*p=0.000*
Limited English (%)	1.70	7.53	2.81	3.39	21.31	2.12	2.49
	(2.64)	(6.65)	(3.21)	(4.86)	(9.23)	(2.84)	(4.96)
	p=0.000	*p=0.000*	*p=0.000*	*p=0.000*	*p=0.000*	*p=0.000*	*p=0.000*
Single Parent (%)	8.95	7.53	10.70	20.47	14.95	5.70	3.84
	(3.93)	(6.65)	(5.27)	(8.08)	(6.90)	(3.14)	(3.10)

© 2020 Kolak M et al. *JAMA Network Open.*

	p=0.000	*p=0.000*	*p=0.000*	*p=0.000*	*p=0.000*	*p=0.000*	*p=0.000*
Poverty (%)	17.19	22.36	10.68	38.21	25.60	7.82	15.27
	(7.78)	(15.25)	(7.42)	(12.69)	(11.92)	(5.93)	(12.29)
	p=0.000	*p=0.000*	*p=0.000*	*p=0.000*	*p=0.000*	*p=0.000*	*p=0.000*
Per Capita Income ($)	22,687	36,149	29,757	14,979	16,593	40,105	32,156
	(4,979)	(24,767)	(9,809)	(4,377)	(5,502)	(15,692)	(16,517)
	p=0.000	*p=0.000*	*p=0.000*	*p=0.000*	*p=0.000*	*p=0.000*	*p=0.000*
Unemployment (%)	10.05	9.24	7.77	20.22	12.09	6.42	10.66
	(4.49)	(4.95)	(3.86)	(8.29)	(5.14)	(3.00)	(7.12)
	p=0.000	*p=0.000*	*p=0.000*	*p=0.000*	*p=0.000*	*p=0.000*	*p=0.000*
Medically Uninsured (%)	15.24	13.72	11.94	18.54	30.01	8.07	12.75
	(6.31)	(8.20)	(6.79)	(7.37)	(9.11)	(5.13)	(8.12)
	p=0.000	*p=0.000*	*p=0.000*	*p=0.000*	*p=0.000*	*p=0.000*	*p=0.000*
Renters (%)	27.77	71.47	28.59	60.49	52.28	26.43	28.77
	(13.39)	(17.19)	(19.20)	(17.14)	(21.32)	(17.80)	(28.77)
	p=0.000	*p=0.000*	*p=0.000*	*p=0.000*	*p=0.000*	*p=0.000*	*p=0.000*
Rent Burden (%)	12.87	29.14	10.50	20.04	15.09	16.89	18.01
	(7.08)	(22.26)	(8.55)	(14.34)	(13.00)	(9.90)	(11.37)
	p=0.000	*p=0.000*	*p=0.000*	*p=0.000*	*p=0.002*	*p=0.000*	*p=0.000*
Crowded Housing (%)	2.15	5.31	2.61	3.98	15.35	1.34	1.31
	(2.11)	(5.83)	(2.82)	(3.79)	(9.49)	(1.83)	(1.99)
	p=0.000	*p=0.000*	*p=0.000*	*p=0.000*	*p=0.000*	*p=0.000*	*p=0.000*
No Vehicle (%)	6.62	33.27	3.66	25.01	12.24	5.09	10.45
	(4.66)	(22.76)	(3.63)	(14.72)	(10.99)	(4.50)	(12.17)
	p=0.000	*p=0.000*	*p=0.000*	*p=0.000*	*p=0.000*	*p=0.000*	*p=0.005*
PC1 Index	0.36	-0.97	0.64	-3.29	-4.19	1.90	1.49
(SE Advantage)	(1.20)	(2.41)	(1.45)	(1.66)	(2.06)	(1.08)	(1.97)
	p=0.000	*p=0.000*	*p=0.000*	*p=0.000*	*p=0.000*	*p=0.000*	*p=0.000*
PC2 Index	-0.81	-0.31	1.19	-1.31	1.66	0.38	-2.90
(ED Mobility)	(0.73)	(1.13)	(0.71)	(1.22)	(1.06)	(0.72)	(1.60)
	p=0.000	*p=0.000*	*p=0.000*	*p=0.000*	*p=0.000*	*p=0.000*	*p=0.000*
PC3 Index	-0.72	2.89	-0.62	-0.35	0.22	0.48	0.66

© 2020 Kolak M et al. *JAMA Network Open.*

(UR Opportunity)	(0.55)	(1.25)	(0.59)	(0.95)	(1.14)	(0.65)	(1.17)
	p=0.000	*p=0.000*	*p=0.000*	*p=0.000*	*p=0.000*	*p=0.000*	*p=0.000*
PC4 Index	-0.38	0.70	0.56	1.51	-1.63	0.06	-1.81
(MC Accessibility)	(0.53)	(1.01)	(0.58)	(0.97)	(1.05)	(0.52)	(1.05)
	p=0.000	*p=0.000*	*p=0.000*	*p=0.000*	*p=0.000*	*p=0.000*	*p=0.000*

© 2020 Kolak M et al. *JAMA Network Open*.

eReferences.

1. Kind AJ, Jencks S, Brock J, et al. Neighborhood socioeconomic disadvantage and 30-day rehospitalization: a retrospective cohort study. *Ann Intern Med*. 2014;161(11):765-774. Medline:25437404 doi:10.7326/M13-2946
2. Folch DC, Arribas-Bel D, Koschinsky J, Spielman SE. Spatial variation in the quality of American Community Survey estimates. *Demography*. 2016;53(5):1535-1554. Medline:27541024 doi:10.1007/s13524-016-0499-1
3. Spielman SE, Folch D, Nagle N. Patterns and causes of uncertainty in the American Community Survey. *Appl Geogr*. 2014;46:147-157. Medline:25404783 doi:10.1016/j.apgeog.2013.11.002
4. US Centers for Disease Control and Prevention; Agency for Toxic Substances and Disease Registry; Geospatial Research, Analysis, and Services Program. Social Vulnerability Index 2014 [database online]. https://svi.cdc.gov/data-and-tools-download.html. Updated May 19, 2016. Accessed January 1, 2018.
5. Anselin L, Syabri I, Kho Y. GeoDa: an introduction to spatial data analysis. *Geogr Anal*. 2006;38(1):5-22. doi:10.1111/j.0016-7363.2005.00671.x

© 2020 Kolak M et al. *JAMA Network Open.*
